# Stimuli-Responsive Hydrogels in Food Sector: Multi-Component Design, Stimulus-Response Mechanisms, and Broad Applications

**DOI:** 10.3390/gels12030233

**Published:** 2026-03-12

**Authors:** Zhiqing Hu, Rui Zhao, Feiyao Wang, Lili Ren, Liyan Wang, Longwei Jiang

**Affiliations:** 1 College of Engineering and Technology, Jilin Agricultural University, Changchun 130118, China; zhiqinghu@jlau.edu.cn (Z.H.); 15643276737@163.com (R.Z.); 2 College of Food Science and Engineering, Jilin Agricultural University, Changchun 130118, China; wangfeiyao1215@163.com; 3 Key Laboratory of Bionic Engineering (Ministry of Education), College of Biological and Agricultural Engineering, Jilin University, Changchun 130022, China; liliren@jlu.edu.cn; 4 School of Food and Nutrition, Anhui Agricultural University, Hefei 230036, China

**Keywords:** hydrogels, stimuli-responsive, multi-component, applications

## Abstract

Hydrogels are endowed with exceptional hydrophilicity and biocompatibility by their network structure, while also exhibiting soft physical properties similar to living tissues, which renders them ideal biomaterials. Responsive hydrogels—particularly those constructed from multicomponent systems including proteins, polysaccharides, peptides, and polyphenols—have emerged as a frontier research focus owing to their tunable responsiveness and controllable functional properties. In this review, hydrogel response mechanisms were categorized according to pH, ionic strength, temperature, light, enzymes, and multi-stimuli interactions. Key preparation strategies, encompassing chemical, physical, and enzymatic crosslinking, were systematically introduced. The preparation of hydrogels from various food-grade matrices, such as polysaccharide-based, protein-based, peptide-based, and polyphenol-based systems, was also summarized, with emphasis placed on how their tailored structures govern functional performance. Furthermore, innovative applications of responsive hydrogels were highlighted, including targeted delivery of nutrients and bioactive substances (e.g., probiotics, anthocyanins, vitamins) in functional foods, smart packaging and sensing for real-time freshness monitoring of meat and fruits, food quality detection through colorimetric and photothermal sensors, and 4D food printing for personalized nutrition and dysphagia-friendly foods.

## 1. Introduction

Hydrogels are three-dimensional structures composed of hydrophilic polymer networks that can retain large amounts of water within their porous structures through hydration and capillary forces while maintaining structural integrity [1]. In the food industry, hydrogels are primarily formulated using natural, food-grade biopolymers, such as proteins (e.g., whey protein, soy protein, gelatin and casein) and polysaccharides (e.g., alginates, carrageenan, starch and chitosan). This is due to their biocompatibility, biodegradability, and Generally Recognized as Safe (GRAS) status [2,3]. Through various preparation techniques, these materials can be processed into diverse macroscopic and microscopic forms (e.g., bulk gels, films, microbeads, microgels). The technical scope spans traditional methods like ionogelation, thermal setting, and solvent casting to more advanced approaches such as emulsion templating, electrospinning, and additive manufacturing (3D/4D printing) [4,5].

Owing to water-rich, soft-solid properties and tunable physicochemical characteristics, hydrogels have been extensively explored in food applications. Serving as key functional ingredients and systems, they are utilized for texture modification, fat replacement, moisture retention, and the encapsulation and controlled release of bioactive compounds (nutrients, probiotics, antioxidants) [6]. In recent years, scientific and industrial interest has sharply shifted toward developing stimulus-responsive hydrogels. These advanced materials exhibit predictable and often reversible changes in microstructure, swelling state, mechanical strength, or permeability in response to specific environmental triggers such as pH, temperature, ionic strength, light, enzymes, or specific biomolecules [7,8].

Food matrices and the human gastrointestinal tract itself provide a rich, dynamically stimulating environment (e.g., pH gradients during digestion, temperature fluctuations during processing, enzyme activity, microbial metabolites). Leveraging these triggers enables functions such as site-specific delivery of nutrients within the gut, real-time visual monitoring of food freshness, on-demand release of preservatives in active packaging, and the creation of dynamically evolving 4D-printed foods [9,10]. Pioneering research has demonstrated this potential, showcasing hydrogels that release antimicrobial agents at specific pH levels associated with corruption, change color in response to microbial metabolites, or protect probiotics during gastric transit for targeted intestinal release [11,12].

This review aims to provide a comprehensive overview of stimulus-responsive hydrogels designed for food systems. Firstly, hydrogels are categorized based on their primary response mechanisms (pH/ions, temperature, light, enzymes, glucose, and multi-stimuli), elucidating their fundamental physicochemical principles. Secondly, it introduces key preparation methods, covering chemical, physical, and enzymatic crosslinking strategies, as well as hydrogel construction based on diverse food-related matrices, including polysaccharides, proteins, peptides, and polyphenols. Subsequently, applications of stimulus-responsive hydrogels in smart nutrient/bioactive delivery, smart packaging and sensing, food quality monitoring, and personalized nutrition via 4D printing are discussed. Finally, current challenges and future prospects are analyzed, emphasizing pathways toward scalable, robust, and safe implementation of stimulus-responsive hydrogels in the food industry.

This review is intended for researchers in food science and engineering, particularly those working on hydrocolloids, active packaging, and nutrient delivery systems. It aims to provide a systematic and critical overview of the design principles, response mechanisms, and food-related applications of stimuli-responsive hydrogels. By integrating fundamental physicochemical concepts (e.g., Flory–Huggins theory, Donnan equilibrium, and free volume theory) with practical material design strategies, this review seeks to bridge the gap between polymer physics and food material science. The ultimate goal is to equip readers with a mechanistic understanding that enables rational design of food-grade responsive hydrogels for real-world applications—from smart packaging to controlled nutrient release—while highlighting the challenges and opportunities for translating laboratory innovations into industrial practice.

## 2. Classification and Potential Mechanisms of Stimulus-Responsive Hydrogels

**Figure 1:**
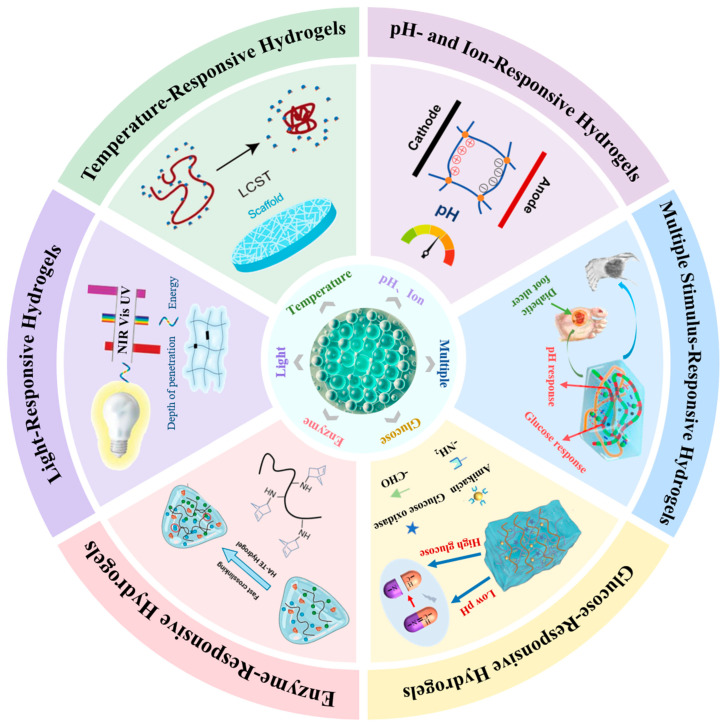
Classification of responsive hydrogels relevant to the food sector, including those responding to pH and ionic strength, temperature, light, enzymes, glucose, and multi-stimuli. Adapted from [13,14,15,16]. All original works are licensed under CC BY 4.0 (https://creativecommons.org/licenses/by/4.0/).

Stimulus-responsive hydrogels can convert environmental stimuli (such as pH, ionic strength, temperature, light, enzymes, etc.) into predictable, programmable changes in their microstructure and/or physicochemical properties. These changes include swelling/deswelling, sol–gel transitions, mechanical property regulation, dynamic crosslinking rearrangement, and controlled release of encapsulated substances. Figure 1 shows the classification of responsive hydrogels relevant to the food sector.

### 2.1. pH- and Ion-Responsive Hydrogels

pH- and ion-responsive hydrogels undergo changes based on the pH value or ionic composition of the surrounding solution, thereby altering the electrostatic interactions between polymer chains forming their internal network [9,17]. The pH determines the charge state of any ionizable side groups on the polymer chains, such as COOH ↔ COO^−^ + H^+^ or NH_3_^+^ ↔ NH_2_ + H^+^. The ionic composition determines the magnitude and range of electrostatic interactions through ion binding and electrostatic screening effects.

The characteristic of pH-responsive hydrogels is that changes in pH alter the ionization state of functional groups within the polymer backbone or crosslinking structure. This induces shifts in electrostatic interactions, osmotic pressure, and water absorption capacity, collectively determining the swelling or shrinkage behavior of the hydrogel network [18,19].

pH-responsive hydrogels can be categorized into three types: anionic, cationic and zwitterionic. Anionic hydrogels—such as poly(acrylic acid) (PAA), alginate, and carboxymethyl cellulose (CMC)—typically contain weakly acidic ionizable groups, such as carboxyl (-COOH) or sulfonic (-SO_3_H) groups, on their polymer chains. At a relatively low pH (pH < pKa), these groups remain largely protonated and unionized, resulting in weak electrostatic repulsion between chains and thus a shrunken hydrogel state. When the pH increases (pH > pKa), the acidic groups become ionized, generating stronger electrostatic repulsion that leads to hydrogel swelling. The key functional groups of cationic pH-responsive hydrogels—such as chitosan, poly(allylamine), and polyethylenimine (PEI)—are amines (e.g., -NH_2_, -NHR, -NR_2_) [17]. Under acidic conditions (low pH), amine groups are protonated and carry a positive charge (-NH_3_^+^), increasing inter-chain electrostatic repulsion and inducing swelling. Under alkaline conditions (high pH), the amines lose their charge, reducing electrostatic repulsion and causing the hydrogel to shrink. In both anionic and cationic hydrogels, the extent of swelling depends on the number of ionizable groups along the polymer chains and their degree of ionization. Generally, a higher density of ionizable groups and a greater degree of ionization lead to more pronounced swelling.

The swelling behavior of zwitterionic (or polyampholyte) pH-responsive hydrogels is significantly more complex than that of their anionic or cationic counterparts. This complexity arises from their unique molecular architecture, where polymer chains bear a balanced distribution of both cationic (e.g., protonated amino -NH_3_^+^, quaternary ammonium) and anionic groups (e.g., carboxylate -COO^−^, sulfonate -SO_3_^−^), maintaining overall charge neutrality at the molecular level [20]. This structural feature implies that both acidic and basic moieties on the polymer backbone can undergo reversible ionization in response to environmental pH changes. Specifically, the swelling exhibits a non-monotonic dependence on pH. Under high pH conditions, the acidic groups (e.g., carboxyl) tend to deprotonate and carry a negative charge (-COO^−^), while the basic groups (e.g., amine) remain neutral (-NH_2_). This results in electrostatic repulsion between chains, leading to hydrogel swelling. Conversely, under low pH conditions, the basic groups become protonated and positively charged (-NH^3+^), while the acidic groups are neutral (-COOH). Again, inter-chain electrostatic repulsion dominates, causing swelling. However, within an intermediate pH range (e.g., near the isoelectric point), both anionic and cationic groups on the polymer chains can be simultaneously ionized, bearing opposite charges. This creates the potential for electrostatic attraction between adjacent chain segments or within the same chain. Such intra- and intermolecular cross-linking effects contract the polymer network, resulting in a shrinkage of the hydrogel volume [21]. Consequently, the ultimate swelling profile of a zwitterionic hydrogel is not only dictated by the environmental pH but is also finely regulated by the type, density, and spatial distribution of the cationic and anionic groups along the polymer backbone.

Ionic strength further modulates hydrogel behavior by screening electrostatic interactions and, in some systems, introducing ion-mediated coordination crosslinks. Multivalent cations such as Ca^2+^ or Fe^3+^ can act as physical crosslinkers, significantly enhancing mechanical strength while retaining environmental responsiveness [22]. In food systems, pH and ionic strength changes during fermentation, spoilage, and digestion provide natural triggers for responsive behavior, supporting applications in intelligent food packaging and digestion-stage-dependent delivery [9,23]. However, the practical performance of pH-responsive hydrogels depends critically on the pKa distribution of ionizable groups—systems with narrow pKa distributions exhibit abrupt volume changes near the pKa, ideal for triggered release, while those with broader distributions show gradual responses suitable for progressive spoilage monitoring [18]. The ionic strength of food matrices also significantly modulates response behavior; high salt concentrations can screen electrostatic interactions and suppress pH-responsive swelling by up to 60% in some systems [22]. Therefore, the design of pH-responsive hydrogels for specific food applications must account for both the target pH range and the ionic composition of the food matrix, which varies considerably between products such as dairy, meat, and plant-based foods.

### 2.2. Temperature-Responsive Hydrogels

Temperature-responsive hydrogels, such as those based on poly(N-isopropylacrylamide) (PNIPAM), hydroxypropyl cellulose (HPC), or gelatin, respond to thermal stimuli through reversible changes in polymer–water interactions. The temperature-responsive behavior of hydrogels arises from a hydrophilic–hydrophobic balance within their polymer networks, leading to a reversible volume phase transition at a critical temperature, typically classified as either an upper critical solution temperature (UCST) or a lower critical solution temperature (LCST) [7].

The LCST-type systems, which undergo collapse upon heating, dominate the research landscape due to their well-defined mechanisms and broad applicability [7,24,25]. The repeating unit of PNIPAM is [-CH2-CH(CONHCH(CH3)2)-]. The side chain contains an amide group and an isopropyl group, creating a hydrophilic-hydrophobic balance. When the temperature exceeds the LCST (~32 °C), the hydrogen bonds between the amide group and water molecules break, and the dominant hydrophobic effect of the isopropyl group prevails, resulting in chain collapse and gel contraction. In contrast, UCST-type hydrogels, which swell upon heating, have been less explored but are gaining interest for their unique properties [26]. The transition temperature of both types can be precisely tuned through molecular design, such as copolymerization with hydrophilic or hydrophobic co-monomers.

The swelling behavior of hydrogels can be quantitatively described by the Flory–Rehner theory, which balances two opposing contributions to the free energy change upon swelling: the mixing free energy (ΔG_mix) and the elastic free energy (ΔG_el) [27,28]. The mixing term follows the Flory–Huggins lattice model, originally developed for polymer solutions [29,30]:

ΔG_mix = RT (n_1_ ln φ_1_ + n_2_ ln φ_2_ + χ n_1_ φ_2_)

where n_1_ and n_2_ are the mole numbers of solvent and polymer, φ_1_ and φ_2_ are their volume fractions, R is the gas constant, T is the absolute temperature, and χ is the Flory–Huggins interaction parameter. The χ parameter quantifies the net interaction energy between polymer segments and solvent molecules: χ = (Δε/kT) · z, where Δε = ε_12_ − (ε_11_ + ε_22_)/2 represents the energy change upon forming a polymer–solvent contact, and z is the lattice coordination number [29,30].

The Flory–Huggins interaction parameter χ is intrinsically linked to solvent quality, which determines the swelling behavior of hydrogels. When χ < 0.5, the solvent is considered “good,” promoting polymer–solvent interactions and favoring hydrogel swelling; when χ > 0.5, the solvent is “poor,” promoting polymer-polymer interactions and leading to network collapse [29]. The temperature dependence of χ arises from changes in hydrogen bonding and hydrophobic interactions. In LCST-type systems (e.g., PNIPAM), heating disrupts hydrogen bonds between polymer and water, increasing χ and driving the transition from good to poor solvent conditions [31]. The volume phase transition temperature (VPTT) corresponds to the point where the net osmotic pressure (π = π_mix + π_el + π_ion) becomes zero [31]. Quantitative analysis of χ parameters for different hydrogel systems reveals values ranging from −0.52 to −0.51 for acrylic acid/gelatin hydrogels under neutral pH conditions, indicating favorable polymer–solvent interactions [32]. The Flory–Rehner framework also explains UCST-type behavior, where χ decreases with temperature, and multi-stimuli responses, where changes in pH or ionic strength alter the effective χ through electrostatic contributions. This thermodynamic perspective provides a unified basis for understanding how molecular-level interactions translate into macroscopic swelling transitions [7].

In food science, temperature-responsive hydrogels based on gelatin (forming gels upon cooling), agar (gelation below ~40 °C), and methylcellulose (heat-induced gelation above ~50 °C), exhibit reversible gelation behavior that can be exploited for texture control and thermal processing stability [9]. These biopolymers undergo phase changes within specific temperature windows, allowing precise modulation of rheological properties, water retention, and structural integrity. For instance, gelatin forms thermally reversible gels at low temperatures, improving the texture of dairy products, desserts, and meats. Methylcellulose derivatives exhibit unique heat-induced gelation, providing shape stability during thermal processing of baked fillings and plant-based products. In addition, temperature-responsive hydrogels can encapsulate and controllably release bioactive compounds such as probiotics, vitamins, and antioxidants. This protects sensitive ingredients during storage and processing and allows triggered release at specific physiological temperatures, enhancing bioavailability [7,33]. Temperature-responsive hydrogels also serve as suitable bio-inks for 3D food printing. Their tailored rheology supports the fabrication of customized food structures with designed texture and nutritional profiles, facilitating personalized nutrition and foods for dysphagia diets [34]. Temperature often acts as a secondary trigger in multi-stimuli-responsive systems. By combining thermal response with sensitivity to pH, enzymes, or ionic strength, advanced hydrogels can achieve more precise, condition-dependent control over food stability and functional release [35].

While temperature-responsive hydrogels offer reversible, tunable responses without chemical additives, their practical application in food systems faces several constraints. Synthetic polymers such as PNIPAM exhibit well-defined LCST behavior but raise regulatory concerns for direct food contact, limiting their use to packaging applications where migration barriers are employed [24]. Food-grade alternatives including gelatin, agar, and methylcellulose are Generally Recognized as Safe (GRAS), but exhibit broader transition temperature ranges and lower mechanical strength compared to synthetic analogs [34]. The gelation temperature of these biopolymers can be tuned through concentration adjustment (e.g., 2–10% *w*/*v* for gelatin) and the addition of cosolutes, but the achievable range (typically 20–60 °C) may not cover all food processing conditions [35]. A fundamental trade-off exists between mechanical strength and responsiveness—increasing crosslinking density to enhance mechanical integrity often reduces the magnitude of the swelling transition. For 3D food printing applications, this balance must be carefully optimized to achieve both printability and post-printing shape changes [34]. Recent interpenetrating network designs have achieved up to 5-fold increases in mechanical strength while retaining >80% of the original swelling ratio, partially addressing this limitation [35].

### 2.3. Light-Responsive Hydrogels

Light-responsive hydrogels enable remote, non-contact, and spatiotemporally precise control over their properties through the adjustment of irradiation parameters, including wavelength, intensity, and duration [36,37]. This controllability originates from the integration of photoactive components into the polymer network. When exposed to light, these photoactive elements, often photochromic molecules, absorb photons and undergo photochemical reactions such as isomerization, bond cleavage, or dimerization [38,39]. The photoactive elements are subsequently translated into macroscopic changes in the hydrogel, including swelling, deformation, or altered mechanical strength [40]. Common response mechanisms encompass photoisomerization, photocleavage, photoinitiated crosslinking, and photothermal conversion, where absorbed light is transformed into heat to activate an embedded thermoresponsive network [36].

Among various light sources, near-infrared (NIR) light-responsive hydrogels has attracted attention due to its superior tissue penetration and lower phototoxicity compared to ultraviolet light, making it suitable for applications requiring remote activation [12,41]. Although much of the early research focused on biomedical applications [42,43], the underlying mechanisms—such as photothermal conversion and photoinduced release—are equally relevant to food systems, where light can serve as a non-invasive trigger for on-demand functions.

In food science, light-responsive hydrogels have been investigated for active packaging and antimicrobial surfaces. Light activation can be harnessed to suppress microbial growth, thereby extending product shelf life and enhancing safety [9,44]. For example, visible light-responsive gels have been fabricated from food-grade biopolymers such as alginate and pectinate, coordinated with Fe (III) ions. This system allows for controlled release profiles upon light exposure, demonstrating potential for packaging applications where on-demand antimicrobial action is desired [45]. A hydrogel scaffold composed of sodium alginate and oligomeric proanthocyanidins was developed. The incorporation of these natural photothermal agents enabled controllable NIR light-responsiveness, suggesting utility in smart packaging where remote thermal activation is beneficial [46,47]. Furthermore, composite hydrogels that encapsulate antimicrobial agents (e.g., plant extracts, silver nanoparticles) have been designed. Upon light irradiation, these systems can trigger the release of encapsulated agents, providing a localized and timed antimicrobial effect for surface coating or padding within food packaging [9,44]. Despite these advantages, challenges related to light penetration, material fatigue under repeated irradiation, and long-term safety considerations remain critical for large-scale implementation [38,48]. Light penetration through food matrices is severely limited—attenuation coefficients for visible light in turbid foods such as milk or meat products can exceed 10 cm^−1^, restricting effective activation to surface layers (<2 mm) [38]. Near-infrared (NIR) light offers improved penetration (up to several centimeters) but requires photothermal conversion agents that must be food-grade and non-migratory [44]. Repeated light exposure can induce fatigue in photochromic molecules, with some systems showing >50% reduction in response magnitude after 10 cycles [48]. Compared to pH or temperature-responsive systems, light-triggered hydrogels are more complex and costly to implement, requiring specialized irradiation equipment. Their niche lies in high-value applications where remote, spatially selective activation justifies the additional complexity, such as intelligent packaging for premium products or on-demand release in sophisticated 4D food printing systems [46].

### 2.4. Enzyme-Responsive Hydrogels

Enzyme-responsive hydrogels undergo structural or property changes upon contact with target enzymes in the environment. This response is mainly achieved through two strategies: (i) embedding bio-polymers susceptible to enzymatic hydrolysis within the hydrogel network (e.g., protease-cleavable proteins, amylase-degradable starch), or (ii) encapsulating substrates that undergo enzymatic conversion to alter hydrogel properties [49,50]. The advantage of enzyme-responsive systems lies in their biological specificity and catalytic efficiency, as enzyme activity exhibits significant variations across different physiological environments, microbial growth stages, or gastrointestinal locations, making them suitable for targeted applications [51]. By introducing enzyme-sensitive dynamic covalent bonds or supramolecular interactions, enzyme stimulation induces progressive softening, pore expansion, or staged depolymerization, thereby enhancing hydrogel behavioral stability and predictability in complex biological environments [50]. These mechanisms are particularly relevant to food systems, where endogenous or microbial enzymes can serve as natural triggers for controlled release and quality monitoring.

In the food industry, enzyme-responsive hydrogels provide a possible platform for “digestion-guided delivery”. Through engineered design, these hydrogels can release encapsulated nutrients, bioactive compounds (e.g., probiotics, vitamins), or antimicrobial agents in a staged manner, responding to the unique enzymatic profiles of different gastrointestinal segments (e.g., pepsin in the stomach, trypsin and amylase in the small intestine) [52]. This approach can enhance bioavailability and enable targeted nutrient absorption. Furthermore, enzyme-responsive hydrogels can be used to control fermentation processes—enzymes dynamically secreted by microorganisms during growth stages serve as natural triggers to modulate the release of fermentation promoters or inhibitors within food matrices [10].

The exquisite specificity of enzyme-responsive hydrogels enables precise targeting of complex biochemical signatures, but also introduces practical limitations. Enzyme activities vary significantly between food products, processing conditions, and storage states—for example, protease levels in different cheese varieties can differ by an order of magnitude [51]. This variability complicates the design of universally applicable systems. Storage stability is a critical concern; pre-exposure to moisture, temperature fluctuations, or microbial enzymes during storage can trigger premature degradation, compromising product shelf life. For protease-sensitive hydrogels designed for digestion-guided delivery, gastric pepsin (0.5–1 mg/mL) typically achieves complete degradation within 2–4 h, while intestinal trypsin (0.1–0.5 mg/mL) requires 4–8 h for equivalent breakdown [52]. This differential kinetics enables staged release but requires careful matching of hydrogel crosslinking density to expected enzyme exposure times. Cost remains a significant barrier—enzyme production and purification add 20–50% to material costs compared to chemically crosslinked analogs, limiting adoption to premium applications [51].

### 2.5. Glucose-Responsive Hydrogels

Glucose-responsive hydrogels are smart systems capable of producing reversible responses to changes in environmental glucose concentration.

The fundamental principle of glucose-responsive hydrogels involves translating glucose recognition into alterations in crosslinking density, hydrophilic-hydrophobic balance, or osmotic pressure within the polymer network, which in turn triggers changes in hydrogel properties such as swelling/shrinking, gel-sol transitions, porosity, and mechanical behavior [53]. Glucose-responsive hydrogels primarily include two types: direct recognition and enzyme-mediated indirect recognition [54]. Directly responsive glucose-responsive hydrogels are represented by phenylboronic acid (PBA) and its derivatives. Through the formation of reversible borate bonds between PBA and glucose, the number of effective crosslinking points and hydrophilicity in the network dynamically change, leading to swelling/shrinkage or alterations in viscoelastic properties. The binding capacity of PBA dependents on its ionization state. Consequently, the response sensitivity and reversibility of this hydrogel system at target pH conditions can be optimized through molecular structural modification, copolymer monomer incorporation, or microenvironmental regulation [55]. Enzyme-mediated indirect glucose-responsive hydrogels are centered on glucose oxidase (GOx) catalysis. GOx oxidizes glucose to gluconic acid, simultaneously producing H_2_O_2_ and consuming O_2_. This reaction amplifies the glucose signal and converts it into changes in pH, oxygen concentration, or oxidative stress, which subsequently trigger swelling, degradation, or controlled release within the hydrogel network [56].

The mechanisms of glucose-responsive—both direct recognition (e.g., PBA-based) and enzyme-mediated (e.g., GOx-based)—are particularly valuable for food analysis, where glucose concentration serves as an indicator of food quality, ripening, or microbial contamination. For instance, by leveraging the hydrogel’s specific binding affinity for sugars, capacitance measurements enable rapid differentiation between various sugar types (such as glucose, fructose, and sucrose) and can even distinguish between different brands of apple juice [57]. Additionally, a previous study investigated DNA-based hydrogel aptamer sensors for detecting *Vibrio parahaemolyticus*. In the presence of target bacteria, the hydrogel network dissociates and releases encapsulated invertase, which catalyzes sucrose conversion to glucose, enabling quantitative detection via a portable glucose meter [58].

While glucose-responsive hydrogels offer unique capabilities for sugar sensing, the two main mechanisms have distinct limitations for food applications. PBA-based systems provide reversible, non-enzymatic glucose recognition but suffer from limited selectivity—PBA binds fructose and galactose with comparable affinity (binding constants: glucose 10–100 M^−1^, fructose 50–500 M^−1^) [55]. In complex food matrices containing multiple sugars, this cross-reactivity leads to ambiguous responses. The pH dependence of PBA binding (optimal at pH > pKa, typically 8–9) also mismatches the acidic to neutral pH range of most foods (pH 3–7) [55]. GOx-based systems offer higher specificity but consume oxygen and produce hydrogen peroxide, which can oxidize sensitive food components [56]. Response times are typically slower (30–60 min) compared to PBA-based systems (5–15 min), and oxygen dependence limits functionality in anaerobic environments such as packaged foods [56]. The choice between mechanisms for a given food application must balance selectivity, response time, pH compatibility, and matrix effects.

### 2.6. Multiple Stimulus-Responsive Hydrogels

Single stimulus-responsive hydrogels often face limitations when operating within complex, multi-signal environments, such as those encountered during food processing (e.g., fermentation) or after consumption in the gastrointestinal tract. In food science, the gastrointestinal tract is studied as a relevant physiological environment where food components are digested and absorbed; understanding how hydrogels respond to its dynamic conditions (e.g., pH gradients, enzyme activities) is essential for designing functional foods that deliver nutrients and bioactive compounds with enhanced bioavailability. To overcome the limitations of single-stimulus systems, multi-stimuli-responsive hydrogels have been engineered by integrating two or more distinct response mechanisms (e.g., temperature, pH/enzyme, redox/enzyme, pH/light, or magnetic/thermal coupling) [13,59]. This integration endows hydrogels with enhanced selectivity, robustness, and functional precision, enabling them to respond to multiple external triggers either simultaneously or in a sequential manner.

The gastrointestinal tract presents a naturally occurring sequence of stimuli, including shifting pH values and region-specific enzyme activities. A hydrogel may remain stable in the acidic stomach (pH-resistant) but swell and degrade in the neutral-to-alkaline small intestine upon encountering specific pancreatic enzymes (e.g., trypsin or amylase). This pH/enzyme dual-response strategy is valuable for intestinal delivery of food bioactives. Research has demonstrated its effectiveness in protecting probiotics, vitamins, or bioactive peptides during gastric transit and ensuring their targeted release in the intestinal region for optimal absorption [52]. More sophisticated systems incorporate a third trigger, such as time or microbial metabolites, to achieve even more precise delivery, which is useful for delivering prebiotics or nutraceuticals intended to modulate the gut health [60]. In fermentation, parameters like pH, sugar concentration (detectable via enzymatic methods), and temperature undergo dynamic changes. pH/enzyme- or temperature/enzyme-responsive hydrogels can be used to autonomously regulate the fermentation process. For example, a hydrogel could encapsulate a nutrient or an antimicrobial agent. When pH decreases (indicating active fermentation) and amylase is present (signaling sugar depletion), the hydrogel releases nutrients to promote microbial growth. Conversely, if temperature exceeds the optimal range, the hydrogel releases antimicrobial agents to prevent spoilage. The hydrogel functions as a self-regulating system capable of responding to the complex chemical signatures of the fermentation state [61,62].

However, the response of dual-responsive hydrogels to combined stimuli can be classified as additive, synergistic, or antagonistic, depending on the coupling between mechanisms. Analysis of Flory–Huggins parameters under combined pH and temperature changes reveals that the coupling term can account for up to 20% of the total response in systems where ionization affects hydrophobic interactions [6]. Practical implementation requires careful consideration of trigger sequence—pH-responsive swelling typically occurs within minutes, while enzyme degradation requires hours, enabling staged responses to sequential stimuli such as gastric-to-intestinal transit [62]. Despite their promise, multi-responsive hydrogels face challenges in reproducibility, with batch-to-batch variations in response profiles of 10–30% commonly reported [13], complicating standardization for food applications requiring regulatory approval.

## 3. Fabrication of Stimulus-Responsive Hydrogels

Stimulus-responsive hydrogels are primarily fabricated through three classes of methods: chemical crosslinking, physical crosslinking, and enzymatic crosslinking. The choice of method dictates key network properties such as mechanical strength, reversibility, and responsiveness. For food applications, where safety, scalability, and precise functionality are paramount, understanding how each technique dictates the final network properties—such as mesh size, mechanical strength, swelling kinetics, and the incorporation of responsive motifs—is crucial [9]. This section focuses on how each technique is employed to construct hydrogels with tailored stimuli-responsive behaviors for food-related applications.

### 3.1. Chemical Crosslinking

**Figure 2:**
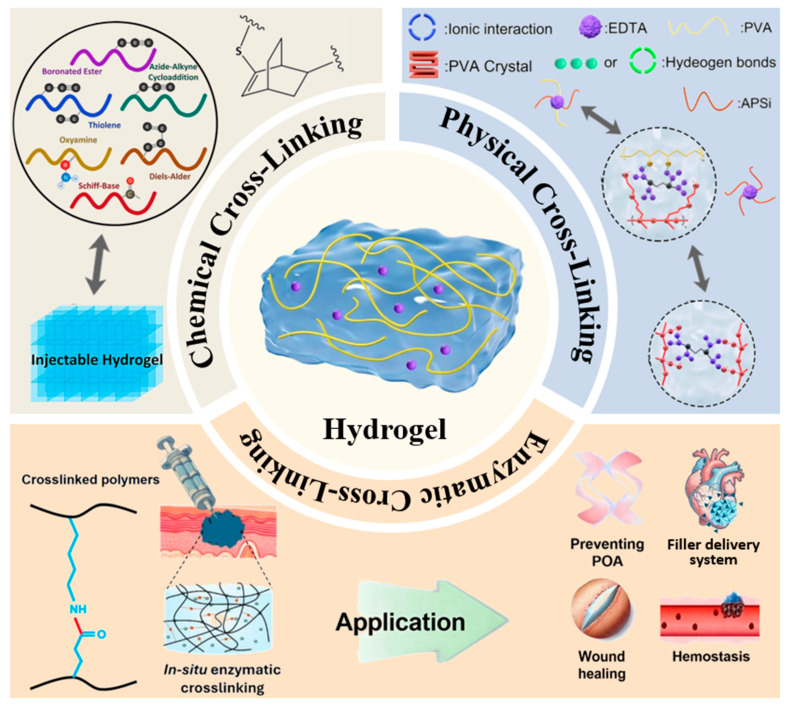
Schematic diagram of hydrogels preparation using chemical crosslinking, physical crosslinking, and enzymatic crosslinking. Adapted from [64,65,66]. All original works are licensed under CC BY 4.0 (https://creativecommons.org/licenses/by/4.0/).

Chemical crosslinking creates permanent covalent bonds between polymer chains, providing structural stability. The value of chemical crosslinking in creating responsive hydrogels lies in its ability to precisely integrate specific functional monomers into the polymer backbone, enabling programmable responses to environmental triggers. Recent advances in nanocomposite hydrogels have further expanded the design space by incorporating nanoparticles into chemically crosslinked networks, imparting additional functionalities such as optical or electrical responsiveness [63]. Figure 2 shows a schematic diagram of hydrogel preparation using chemical crosslinking, physical crosslinking, and enzymatic crosslinking.

#### 3.1.1. Free Radical Polymerization

Free radical polymerization is one of the most commonly used chemical methods for synthesizing stimuli-responsive hydrogels [9]. The responsive behavior of hydrogels prepared by free radical polymerization can be precisely tuned by adjusting monomer ratios and crosslinker concentrations. For instance, the lower critical solution temperature (LCST) of poly(N-isopropylacrylamide)-based hydrogels can be modulated across a range of 25–40 °C by copolymerizing with hydrophilic or hydrophobic comonomers, enabling customization for specific food processing temperatures [24,25]. The crosslinking density, determined by the concentration of crosslinkers such as N,N’-methylenebisacrylamide, directly affects the swelling ratio and mechanical strength; increasing crosslinker concentration from 0.5 to 2.0 mol% has been shown to decrease equilibrium swelling by approximately 40% while enhancing compressive modulus from 10 kPa to 45 kPa [67]. These tunable parameters allow the design of hydrogels with targeted responsiveness for applications such as controlled release in fruit packaging. For instance, pH/temperature-responsive hydrogel films constructed from TEMPO-oxidized nanofibrillated cellulose (TOCNFs) and cationic-modified poly(N-isopropyl acrylamide-co-acrylamide) (CPNIPAM-AM) enabled controlled release of the preservative natamycin triggered by the microenvironment within fruit, thereby facilitating active climacteric fruit packaging [68].

#### 3.1.2. Graft Copolymerization

Graft copolymerization is a widely employed strategy for designing responsive hydrogels, particularly those made from food-grade polymers. Functional side chains are chemically bonded to the polymer backbone, introducing responsive groups that enable the hydrogel to react to external triggers such as temperature, pH, light, or ionic strength. Beyond imparting stimulus sensitivity, grafting can also modulate network porosity and other physicochemical properties [69]. This technique provides a versatile route for introducing hydrophobic segments, charged groups, or other sensitive units into natural polymers, facilitating the development of hydrogels with tailored responsiveness for food applications. For instance, grafting butyl glycidyl ether onto soluble starch followed by CDT group modification yielded hydrogels exhibiting dual temperature and pH responsiveness, demonstrating potential for smart food systems and controlled-release applications [70].

#### 3.1.3. Radiation-Induced Crosslinking

Radiation-induced crosslinking utilizes high-energy radiation (e.g., γ-rays, electron beams) or light to generate reactive species that directly initiate polymerization, crosslinking, or grafting, forming hydrogel networks without chemical initiators [71]. This technique offers precise control over network structure through radiation dose, which directly determines crosslinking density, mesh size, and swelling behavior.

For temperature-responsive hydrogels prepared via electron beam irradiation, study indicates that increasing radiation dose from 10 to 50 kGy progressively decreases the volume phase transition temperature by 3–5 °C and reduces equilibrium swelling by 25–35%, attributed to higher crosslinking density restricting chain mobility [72]. The competition between polymer chain scission and crosslinking, influenced by radiation dose and monomer concentration, exerts a crucial effect on the hydrogel’s final phase transition temperature, swelling kinetics, and porous microstructure.

Beyond static networks, radiation-induced crosslinking can produce self-healing hydrogels through the formation of reversible dynamic bonds. Healing efficiency of up to 85% has been achieved at optimal radiation doses of 25 kGy [73]. Such tunable properties enable the design of hydrogels for specific food packaging applications requiring controlled swelling and self-repairing capabilities.

### 3.2. Physical Crosslinking

Physical crosslinking utilizes non-covalent interactions such as hydrogen bonds, hydrophobic interactions, and electrostatic interactions to assemble polymer networks into reversible, stimuli-responsive hydrogels. The dynamic nature of these bonds endows the materials with injectability, self-healing, and tunable responsiveness to environmental triggers (e.g., temperature, pH, ionic strength).

#### 3.2.1. Hydrogen Bonds

Cooperative hydrogen bonding between polymer chains serves as dynamic physical crosslinking points, providing hydrogels with structural stability and reversibility [74]. The strength of hydrogen bonds is sensitive to temperature, pH, and ionic strength, making them ideal for constructing stimuli-responsive systems. For gelatin-based hydrogels, increasing concentration from 2% to 10% (*w*/*v*) raises the gel melting temperature from 28 °C to 34 °C and increases storage modulus by approximately one order of magnitude [75]. Similarly, for methylcellulose hydrogels, the sol–gel transition temperature can be tuned between 40 °C and 60 °C by adjusting polymer concentration and salt addition, enabling precise control over thermal gelation behavior for food processing applications [72,73,76].

#### 3.2.2. Hydrophobic Interactions

Hydrophobic interactions contribute to hydrogel formation by driving the aggregation of nonpolar segments in aqueous environments, typically achieved through micellar copolymerization where hydrophobic blocks are encapsulated within surfactant micelles [77,78]. Under external forces, micelles effectively disperse stress while the slippage and disentanglement of hydrophobic chains dissipate significant energy, thereby greatly improving the toughness of hydrogels [79]. For instance, a polyacrylamide hydrogel reinforced with phenylboronic acid (PBA) latex particles, using sodium dodecyl sulfate (SDS) and gum arabic (GA) as composite surfactants, exhibited enhanced fracture stress and toughness due to synergistic hydrogen bonding between GA and the polymer network [80]. A hydrophobic associative hydrogel constructed with SDS and the zwitterionic surfactant dodecyl dimethyl betaine achieved a tensile stress of ~690 kPa, excellent fatigue resistance and room-temperature self-healing capability [81]. Such materials hold promise for reusable food packaging or self-repairing edible films.

#### 3.2.3. Electrostatic Interactions

Electrostatic interactions are a key mechanism for physical crosslinking in polyelectrolyte hydrogels. Ionic crosslinking—where polyanions (e.g., alginate, pectin) bind multivalent cations (Ca^2+^, Fe^3+^)—allows rapid gelation under mild conditions, with crosslinking density tunable by ion concentration [82,83]. For example, hybrid ion-hydrogen bond crosslinking between Fe^3+^ and sodium alginate/poly(acrylamide-co-acrylic acid) networks produced hydrogels with high stiffness, toughness, and self-healing properties that remain stable across a wide pH range and in saline environments [84]. Another form, polyelectrolyte complexation between oppositely charged polymers (e.g., chitosan and alginate), yields pH-responsive gels whose swelling and dissociation behavior can be modulated by environmental pH, relevant for targeted nutrient release or smart packaging [85]. These electrostatic principles are exploited in food applications such as encapsulation of probiotics, texture control in dairy products, and moisture regulation in edible coatings.

### 3.3. Enzyme Crosslinking

Enzyme crosslinking utilizes specific enzymes (such as horseradish peroxidase, tyrosinase, transglutaminase, etc.) as catalysts to form covalent bonds between functional groups on polymer chains [86,87].

Horseradish peroxidase (HRP), in the presence of hydrogen peroxide (H_2_O_2_), catalyzes the oxidation of phenolic compounds to generate reactive radicals that couple to form crosslinks. As reviewed by Li et al., peroxidase (including HRP) can catalyze the oxidation of food molecules (proteins, polysaccharides, polyphenols) in two steps: an enzymatic reaction generating free radicals or quinones, followed by non-enzymatic coupling with other molecules [86]. Polymers bearing phenolic groups can serve as substrates for HRP-mediated crosslinking, which has potential applications in constructing food colloidal systems such as emulsions, nanoparticles, and microgels for encapsulating and delivering bioactive compounds [86].

Tyrosinase is a copper-containing enzyme that oxidizes phenolic and catecholic substrates to reactive orthoquinones, which subsequently undergo non-enzymatic coupling with amino or thiol groups, forming crosslinked networks [88,89]. Tyrosinase has been directly applied in food systems. A previous study systematically compared the effects of tyrosinase, laccase, and transglutaminase on goat milk Halloumi-type cheese production [87]. Results showed that tyrosinase treatment significantly improved cheese yield and quality, with notably enhanced sensory attributes including brighter appearance and intensified milk flavor. The study confirmed the formation of protein polymers and increased β-sheet content in tyrosinase-treated cheese. Additionally, tyrosinase has been investigated for modifying dairy proteins. A research demonstrated that mushroom tyrosinase could crosslink whey protein isolate with alginic acid to form stable gels, with optimal viscous properties achieved at specific enzyme concentrations and incubation times [90]. Tyrosinase was also employed to construct edible composite films using pea protein, chitosan, and chlorogenic acid [91]. At optimal conditions (PP:CS ratio 1:1, 20 mmol/L CA, 18 U/mL Tyr), the composite film exhibited excellent mechanical properties (tensile strength 130.78 MPa), antioxidant capacity (DPPH scavenging 69.05%), and water vapor barrier performance.

The choice of crosslinking method depends on the desired stimuli-responsive behavior, material compatibility, and target food application. Chemical crosslinking (e.g., free radical polymerization, radiation-induced crosslinking) produces permanent covalent networks with high mechanical strength and stability, making it suitable for applications requiring robust structures such as reusable food packaging films or long-term encapsulation matrices [71,92]. However, the use of chemical initiators or radiation may limit compatibility with sensitive bioactive compounds. Physical crosslinking, based on reversible non-covalent interactions (hydrogen bonds, hydrophobic associations, electrostatic interactions), offers mild gelation conditions and stimuli-responsive reversibility. This approach is particularly advantageous for fabricating injectable or self-healing hydrogels for applications such as edible coatings, probiotic encapsulation, and temperature-responsive texture modifiers [74,77,82]. The reversibility of physical crosslinks enables dynamic responses to environmental changes, but may result in lower mechanical strength compared to chemically crosslinked networks. Enzymatic crosslinking provides a biocompatible alternative under mild aqueous conditions, enabling the incorporation of heat-sensitive nutrients and bioactive compounds [93]. This method is especially suitable for food applications requiring precise control over gelation kinetics and network structure, such as plant-based protein structuring, dairy product modification, and targeted nutrient delivery [94,95]. The substrate specificity of enzymes allows for selective crosslinking of proteins and polysaccharides, but the cost and scalability of enzyme production remain considerations for industrial implementation. In practice, hybrid crosslinking strategies combining two or more methods are increasingly employed to achieve synergistic properties. For example, combining ionic crosslinking with enzymatic crosslinking can produce hydrogels with enhanced mechanical strength and tunable responsiveness [84,96]. The selection of the most suitable method ultimately requires balancing the requirements of mechanical performance, response characteristics, biocompatibility, and processing conditions for the specific food application.

Table 1 summarizes the key formulation parameters for various hydrogel systems discussed in this section, along with their optimal ranges and corresponding performance outcomes.

## 4. Construction of Different Matrix Stimulus-Responsive Hydrogels

**Figure 3:**
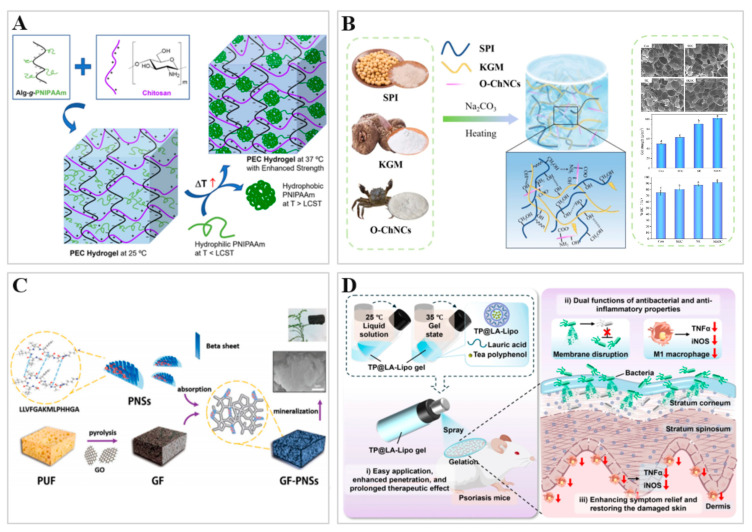
Schematic preparation of hydrogels based on different matrices: (**A**) polysaccharide-based. Adapted from [98]; (**B**) protein-based. Quoted from [99]; (**C**) peptide-based. Quoted from [100]; (**D**) polyphenol-based hydrogels. Quoted from [101]. All original works are licensed under CC BY 4.0 (https://creativecommons.org/licenses/by/4.0/).

Stimulus-responsive hydrogels in the food sector may be categorized according to their matrix origin into polysaccharide-based, protein-based, peptide-based, and polyphenol-based types. Figure 3 shows the schematic preparation of hydrogels based on different matrices.

### 4.1. Polysaccharide-Based Hydrogel

Polysaccharides are abundant in resources, diverse in structure, and possess excellent biocompatibility and degradability [102]. Natural polysaccharide chains harbour numerous active functional groups, facilitating their modification or further chemical reactions. Consequently, they are extensively employed in the synthesis of stimulus-responsive hydrogels [103]. Chitosan, sodium alginate and cellulose are frequently employed in the preparation of stimulus-responsive hydrogels [104].

Chitosan, composed of β-(1→4)-linked D-glucosamine units, contains reactive amino and hydroxyl groups that confer pH responsiveness [105]. Under acidic conditions (pH < pKa ~6.5), the amino groups protonate to -NH_3_^+^, generating electrostatic repulsion that leads to gel swelling. Under neutral or alkaline conditions, deprotonation reduces repulsion and causes gel contraction. Chitosan has been widely investigated for encapsulating and delivering food bioactive compounds. For instance, Zhong et al. developed a novel pH-responsive hydrogel based on aminoethyl-quaternized chitosan (ONC), ethylenediamine-modified gelatin (Am-Gel), and oxidized sodium alginate (OSA) for oral vitamin B12 (VB12) delivery [106]. The hydrogel exhibited strong pH-dependent swelling behavior, with maximum swelling observed at pH 6.8–7.4. In vitro release assays demonstrated significant pH sensitivity: cumulative VB12 release was below 70% in simulated gastric fluid (SGF, pH 1.2) but approached 100% in simulated intestinal fluid (SIF, pH 6.8) and colonic fluid (SCF, pH 7.4). The release kinetics followed the Peppas-Sahlin model, dominated by Fickian diffusion and modulated by crosslinking density. Additionally, the hydrogel displayed rapid self-healing within 30 s, selective antibacterial activity against Staphylococcus aureus (inhibition zone: 9.5 ± 0.55 mm), and strong antioxidant capacity (DPPH scavenging >60%), highlighting its multifunctional potential for intelligent oral delivery systems in food and pharmaceutical applications [106].

Cellulose is a linear polymer composed of β-D-glucose units linked by β-(1→4) glycosidic bonds. Its molecular chains are rich in hydroxyl groups, readily forming strong hydrogen bonds, conferring excellent mechanical strength and chemical stability [107,108,109]. Cellulose derivatives—such as carboxymethyl cellulose (CMC), hydroxypropyl cellulose (HPC), and methylcellulose—are frequently used to fabricate stimuli-responsive hydrogels for food applications. A review highlights the potential of cellulose-based hydrogels as films, coatings, and indicators for food packaging, owing to their ability to regulate moisture, carry bioactive substances, and respond to environmental stimuli [110]. pH/temperature dual-responsive hydrogel films constructed from TEMPO-oxidised nanofibrillated cellulose (TOCNFs) and cationic-modified poly(N-isopropyl acrylamide-co-acrylamide) (CPNIPAM-AM) enabled the controlled release of the preservatives (such as natamycin) in fruit packaging, effectively extending shelf life [68]. A bilayer hydrogel actuator composed of TEMPO-oxidized cellulose nanofibers/poly(N-isopropylacrylamide) (TOCN/PNIPAM) as the responsive layer and TOCN/polyacrylamide (TOCN/PAM) as the non-responsive layer exhibited excellent mechanical properties (compressive strength ~89.2 kPa, elongation at break ~170.7%, tensile strength ~24.0 kPa) and temperature-driven actuation. The incorporation of Fe^3+^ further endowed the hydrogel with shape memory capability, enabling its use as a temperature-responsive switch for environmental monitoring and flexible sensing applications [111].

Sodium alginate is an anionic polysaccharide β-D-mannuronic acid (M) and α-L-guluronic acid (G) units. The G-block regions are rich in carboxyl groups, enabling ionic crosslinking with divalent cations (e.g., Ca^2+^) to form “egg-box” structures. This ionic crosslinking process—which is rapid, occurs under mild conditions, and requires no toxic reagents—is widely exploited in food applications such as encapsulation of bioactive compounds, texture modification of processed foods, and formation of edible coatings [112]. Alginate-based hydrogels have been extensively studied for encapsulating probiotics, enzymes, and bioactive compounds to enhance their stability during processing and gastrointestinal transit. For instance, thermoresponsive gelatin-alginate hybrid hydrogels were developed as food-grade carriers for controlled release of meat scent molecules in plant-based meat products [113]. The semi-interpenetrating network formed by alginate and gelatin resisted structural degradation at elevated temperatures, enabling sustained scent release during cooking, which represents a practical application in plant-based meat formulation.

### 4.2. Protein-Based Hydrogels

Natural food proteins, including plant and animal proteins are widely employed in hydrogel preparation owing to their favorable gelation properties, nutritional value, and biodegradability [9,114]. Protein-based hydrogels can respond to stimuli such as pH, temperature, enzymes, and ionic strength, enabling applications in food texture modification, bioactive compound delivery, and 3D food printing [115].

Whey protein isolate (WPI) systems, particularly when combined with polysaccharides such as chitosan oligosaccharide or subjected to transglutaminase crosslinking, can be employed to enhance the texture of low-fat dairy products by constructing multi-scale structures such as emulsifying gel micelles and microgels. For instance, incorporating whey protein emulsifying gel micelles into low-fat cheese improves the system’s structural support and mouthfeel smoothness, mitigating issues of thin texture and granularity arising from fat reduction [116]. This microgel-stabilized Pickering emulsion approach effectively mitigated the texture defects associated with fat reduction, producing cheese with denser structure, improved hardness and elasticity, and more uniform fat globule distribution while maintaining desirable flavor profiles [116]. Furthermore, whey protein hydrogels have been employed for stabilizing bioactive compounds such as polyphenols and anthocyanins. The gel network effectively inhibits the migration and oxidation of these active ingredients, thereby enhancing stability during processing and storage [117]. Yu et al. investigated the combined effects of TG crosslinking and ultrasonication on whey protein isolate- chitosan oligosaccharide (WPI-COS) microgel dispersions. They found that moderate unfolding induced by ultrasonication during TG crosslinking significantly improved the interfacial properties of the microgels, including increased surface hydrophobicity, reduced interfacial tension, and enhanced emulsifying performance. Microstructural analysis revealed that ultrasound-induced unfolding led to smaller emulsion droplets, more uniform distribution, and thicker interfacial films at the oil-water interface, demonstrating the feasibility of conformational regulation in tuning the techno-functional features of protein microgels [118].

Soy protein isolate (SPI) has attracted considerable attention for 3D food printing applications. Due to its favorable surface activity and capacity for multiple non-covalent interactions, SPI is frequently combined with polysaccharides to formulate printable inks with tailored rheological properties [119]. For example, the incorporation of Naematelia aurantialba polysaccharide (NAP) into thermally induced SPI gels progressively enhanced rheological parameters (viscosity profiles, thixotropic behavior, creep recovery response), textural attributes, water-holding capacity, and dimensional accuracy during 3D printing in a dose-dependent manner. At the optimal NAP concentration of 2.00% (*w*/*v*), the composite gel exhibited enhanced viscosity and printing fidelity, attributed to synergistic effects of hydrogen bonding, electrostatic attractions, hydrophobic associations, and disulfide linkages between NAP and SPI. Microstructural analysis revealed a denser and more uniform network with increased β-sheet content [120]. SPI-based hydrogels have also been explored for dysphagia-friendly food development. Composite gels incorporating Tremella polysaccharides and psyllium husk powder demonstrated tunable mechanical strength and printing precision, with optimized formulations showing potential for manufacturing swallowable food products with customized textures [121] SPI can form composite gels with polysaccharides such as carrageenan for encapsulating hydrophobic bioactive compounds (e.g., curcumin). Studies on SPI-dextran conjugate hydrogels prepared via Maillard reaction and transglutaminase crosslinking revealed that higher molecular weight dextran improved resistance to gastrointestinal digestion while maintaining a homogeneous gel network, enabling controlled release of encapsulated curcumin [122]. Similarly, SPI-egg white composite microgels fabricated at pH 4 exhibited compact structures resistant to gastrointestinal digestion, achieving sustained curcumin release with preserved antioxidant activity [123]. Employing an emulsified filling gel strategy, where emulsified particles encapsulating enzymatically hydrolysed proteins are introduced as the filling phase into the SPI network, significantly enhanced the gel’s strength, water-holding capacity, and structural integrity. This approach offers an innovative solution for structuring alternative products such as plant-based meat [124].

### 4.3. Peptide-Based Hydrogel

Owing to the molecular structure of peptide-based hydrogels bearing a high degree of similarity to natural biomolecules, they typically exhibit excellent biocompatibility and degradability. Furthermore, peptide-based hydrogels possess a high degree of programmability, allowing their network structure to be modulated by adjusting amino acid sequences or in response to environmental stimuli such as pH or temperature, thereby enabling controlled and sustained release of active substances [125,126]. Peptide-based hydrogels are typically constructed through molecular self-assembly or directed cross-linking [127]. For instance, the ionically self-complementary peptide RADA16-I can form distinct nanofibre scaffolds through molecular self-assembly. This hexadecapeptide forms stable β-sheet structures, which further assemble into nanofibres, ultimately constituting hydrogel scaffolds with over 99.5% water content [128]. Recent advances in food-derived self-assembling peptides have demonstrated that hydrogels constructed from food protein sources (e.g., soybean, whey, collagen) exhibit tunable rheological properties and stimuli-responsive behavior, making them suitable for food texture modification and bioactive compound delivery [125].

Self-assembled peptide hydrogels derived from food sources have shown particular promise in food applications. For example, a Zn^2+^-coordination-driven helical dodecapeptide hydrogel derived from oyster and mussel proteins was developed as a delivery system for food applications, demonstrating the potential of food-derived peptides to form supramolecular hydrogels with tunable network structures [129]. The self-assembly mechanism is governed by non-covalent interactions including hydrogen bonding, hydrophobic interactions, and π-π stacking, which can be modulated by environmental factors such as temperature, pH, and ionic strength [130]. Peptide-based hydrogels serve as carriers for functional food active ingredients, enabling encapsulation and controlled release. For instance, a pH-responsive peptide hydrogel has been employed to encapsulate natural antioxidants such as anthocyanins. This hydrogel maintains stability under gastric acid conditions within simulated digestive environments and facilitates controlled release within the intestinal tract, thereby enhancing the bioavailability of nutrients [131].

Peptide hydrogels possess antimicrobial properties that can inhibit food spoilage and pathogens, while their biodegradability supports eco-friendly practices and sustainable packaging, including edible films and coatings that extend shelf life [130]. A self-assembled antimicrobial peptide hydrogel derived from the fish-derived peptide Pardaxin was developed as a functional material for food packaging and preservation [132]. This hydrogel forms under mild conditions without chemical crosslinkers, exhibits excellent viscoelastic properties and shear-thinning behavior, and demonstrates intrinsic antimicrobial activity against foodborne and drug-resistant pathogens through a ‘trap-and-kill’ mechanism. Digestibility studies confirmed enzymatic degradation of released peptides post-ingestion, enabling tunable antimicrobial function. Practical food applications demonstrated strong adhesion to meat surfaces, protection against *Shewanella putrefaciens*, UV barrier properties, and sustained release in liquid foods such as milk.

### 4.4. Polyphenol-Based Hydrogel

Polyphenols are a class of naturally occurring bioactive compounds rich in phenolic hydroxyl groups, primarily comprising flavonoids (such as catechins and anthocyanins) and non-flavonoids (such as tannic acid and gallic acid) [133]. Their characteristic resorcinol/catechol structure enables them to interact with polymer matrices via coordination bonds, hydrophobic interactions, hydrogen bonds, and π-π stacking, thereby constructing hydrogel systems with multifunctional properties including antioxidant, antibacterial, anti-inflammatory, and metabolic regulation capabilities [134]. Beyond serving as active ingredients, polyphenolic molecules confer intelligent properties—such as self-healing, tissue adhesion, and stimulus responsiveness—through their dynamically reversible cross-linking mechanisms (e.g., hydrogen bonds, coordination bonds). This renders them highly promising for smart food packaging applications [135].

For functional food delivery, polyphenol hydrogels offer novel approaches for efficient delivery of hydrophobic nutrients due to their pH responsiveness and targeted release properties. Zhang et al. designed calcium alginate/chitosan nanocomposite hydrogel beads that achieve intestinal targeted release of fucoxanthin through pH-dependent swelling-shrinking behavior. Release rates were below 3.8% in simulated gastric fluid but reached 99% in intestinal fluid [136]. Hu et al. employed food-grade polysaccharide self-assembly to construct polyelectrolyte composite hydrogels, utilizing dynamic hydrogen bonding and π-π stacking interactions for sustained-release of tea polyphenols, offering an eco-friendly solution for gut-targeted nutrient delivery [137].

Furthermore, polyphenol hydrogels find extensive application in food packaging due to their antimicrobial, antioxidant, preservative, and biodegradable properties. Using carboxymethyl chitosan and oxidized carrageenan as substrates, hydrophobic polyphenols were successfully introduced into multifunctional hydrogel films through borate ester bonds, with mechanical strength further enhanced by Schiff base bonds [138]. The prepared hydrogel films exhibited antibacterial rates exceeding 98% against Escherichia coli and Staphylococcus aureus, and demonstrated excellent antioxidant and UV shielding properties. Banana preservation experiments demonstrated that these hydrogel films effectively delayed fruit deterioration, with the film exhibiting 90% biodegradability in soil within 60 days. Similarly, high strength chitosan-tea polyphenol (CS-TP) films were fabricated via a novel approach combining alkali dissolution regeneration and one-step self-assembly [139]. Strong hydrogen bonding and hydrophobic interactions between chitosan and tea polyphenols enabled substantial polyphenol adsorption onto the film matrix. Pork preservation results indicated that CS-TP films lowered pH, slowed bacterial growth and color change, and decreased lipid oxidation products in fresh pork compared to control samples, thereby prolonging shelf life by 2 days.

Alginate-based edible films have also been developed as delivery systems for green tea polyphenols, demonstrating the versatility of polysaccharide matrices for incorporating bioactive phenolic compounds in food packaging applications [140]. Yin et al. incorporated hydrophobic polyphenols into multifunctional hydrogels via borate ester bonds, achieving over 98% inhibition rates against *Escherichia coli* and *Staphylococcus aureus* while exhibiting excellent antioxidant and UV-shielding capabilities [138]. In banana preservation experiments, this hydrogel effectively delayed fruit spoilage. Fan et al. employed in situ spinning to fabricate a thermosensitive composite hydrogel with a sheath-core structure, achieving a tea polyphenol release rate of 77.8% and radical scavenging efficiencies of 99.2% for ABTS and 46.6% for PTIO [141].

## 5. Application of Stimulus-Responsive Hydrogels in Food

**Figure 4:**
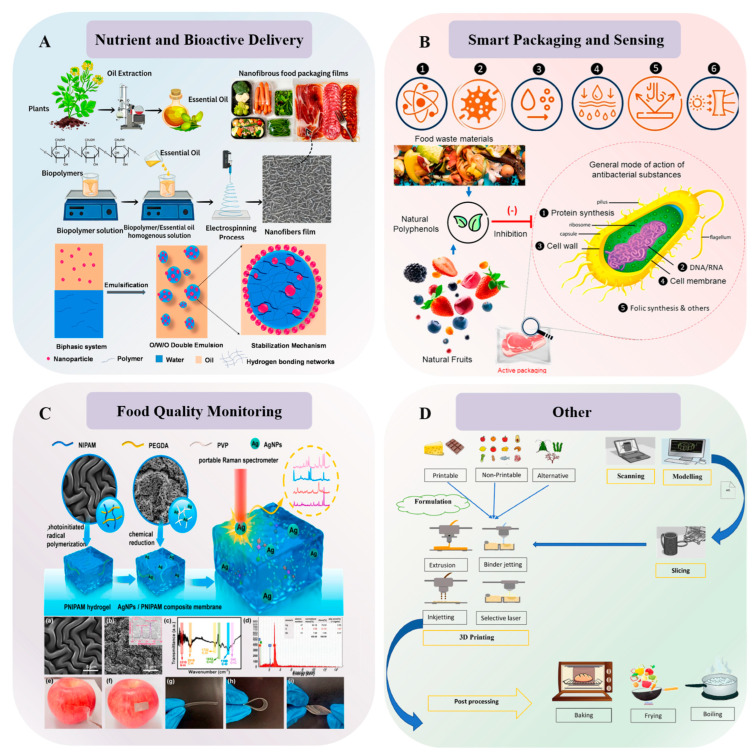
Application of stimulus-responsive hydrogels in food sector: (**A**) nutrient and bioactive delivery. Quoted from [142]; (**B**) smart packaging and sensing. Adapted from [143]. This image is adapted from a work licensed under CC BY 4.0 (https://creativecommons.org/licenses/by/4.0/); (**C**) food quality monitoring. ((**a**) Surface morphology of PNIPAM hydrogel; (**b**) SEM image of AgNP-loaded PNIPAM composite; (**c**) Raman spectrum showing SERS activity on the composite hydrogel; (**d**) Elemental distribution map confirming Ag presence; (**e**,**f**) Visual examples of hydrogel patches applied to apples for direct pesticide detection; (**g**–**i**) Flexibility of the hydrogel film enabling surface adaptability.) Quoted from [144]; (**D**) intelligent foods with 3D/4D printing. Quoted from [145].

Stimulus-responsive hydrogels can be employed to fabricate smart packaging materials that monitor and reflect food quality and safety information, regulate nutrient delivery processes, and detect contaminants in foodstuffs, thereby safeguarding food safety. Figure 4 shows application of stimulus-responsive hydrogels in the food sector.

### 5.1. Nutrient and Bioactive Delivery

Stimulus-responsive hydrogels can encapsulate nutrients and bioactive compounds, enabling controlled release in response to external environmental stimuli such as pH, temperature, or ionic strength. Nutrients primarily encompass proteins, lipids, carbohydrates, vitamins, and minerals. Bioactive compounds including polyphenols and carotenoids, whilst possessing health-promoting potential, frequently encounter challenges such as poor water solubility, low chemical stability, and inadequate bioavailability [47,146]. Through rational design of hydrogel systems, physical isolation and protection can be provided for bioactive substances, thereby enhancing their stability and bioavailability [147].

It is important to distinguish between the two major contexts where stimuli-responsive hydrogels encounter food-related environments: (i) during food processing and storage (e.g., pH changes during fermentation, temperature fluctuations), and (ii) after consumption, when food components are exposed to the gastrointestinal tract. While the latter overlaps with pharmaceutical delivery, the goal in food science is to enhance the bioavailability of nutrients and bioactive compounds, not to achieve therapeutic effects. Therefore, studies on gastrointestinal release are relevant insofar as they inform the design of functional foods with improved health benefits [148,149].

pH-responsive hydrogels, such as chitosan/alginate polyelectrolyte complexes, carboxymethyl cellulose-based gels, and protein-polysaccharide conjugates, have emerged as a prominent area of research for encapsulation and delivery of food bioactives. For instance, a study achieved targeted delivery and sustained release of probiotics within the intestinal environment by constructing a water-in-oil-in-water (W/O/W) emulsion-gel composite system. This system utilised sodium caseinate-carrageenan Maillard reaction products as the emulsifying layer and sodium alginate-carboxymethyl chitosan hydrogel as the shell to encapsulate *Lactobacillus rhamnosus* LR76 [150].

By exploiting the adaptive charge properties of acidic amino acids aspartic acid (D) and glutamic acid (E), two pH-responsive peptide hydrogel carriers, Fmoc-FDFD and Fmoc-FEFE, were designed. These hydrogel carriers effectively encapsulate anthocyanins and enhance their stability in gastric acid environments, ensuring controlled release within the intestinal tract to improve bioavailability [131]. A recent study evaluated yogurt enriched with anthocyanin-loaded nanoliposomes over 21 days of refrigerated storage. The nanoliposome-enriched yogurt exhibited improved color stability, reduced syneresis, and favorable rheological properties compared to free anthocyanin formulations. The anthocyanins maintained strong antioxidant activity (ABTS: 93.24%, FRAP: 1023.24 µM TE/g D.W.) throughout storage, demonstrating that nanoliposomal encapsulation provides effective protection against environmental degradation in dairy matrices [151].

For delivery of peptide-based functional ingredients, a chitosan-sodium alginate-CaCl_2_ system was employed to prepare pH-sensitive hydrogel microspheres encapsulating rice bran active peptide (RBAP). This hydrogel system effectively preserves the structural stability of RBAP during simulated digestion and enables responsive release under varying pH conditions, thereby enhancing its effective delivery within the gastrointestinal environment [152]. Furthermore, Cheng et al. constructed a pH-responsive hydrogel composed of carboxymethyl chitosan/sodium alginate (CMCS/SA) and loaded apigenin as a hydrophobic active compound model. The swelling and release behavior exhibited pronounced pH dependency, with the release process modelled through kinetic analysis, demonstrating potential for encapsulation and protection of hydrophobic nutrients in functional foods [152]. Furthermore, Cheng et al. constructed a pH-responsive hydrogel composed of carboxymethyl chitosan/sodium alginate (CMCS/SA) and loaded apigenin as a hydrophobic active compound model. The swelling and release behavior exhibited pronounced pH dependency, with the release process modelled through kinetic analysis, demonstrating potential for encapsulation and protection of hydrophobic nutrients in functional foods [153].

### 5.2. Smart Packaging and Sensing

Stimulus-responsive hydrogels may be employed in the manufacture of active smart packaging materials. Such materials are typically formulated from natural substances—such as food-grade proteins, polysaccharides, and lipids—to ensure their excellent sustainability, low environmental impact, and low toxicity [154]. Building upon this foundation, smart packaging further emphasizes the provision of readable feedback on food condition. This is achieved through integrated sensing capabilities that reflect key changes in real time during the food deterioration process, including pH fluctuations, accumulation of volatile alkaline compounds and biogenic amines, thereby providing a basis for quality assessment and supply chain management.

A hydrogel based on alginate and methylcellulose, incorporating bromothymol blue as a pH-sensitive dye, was developed as a colorimetric indicator for monitoring minced pork spoilage at 4 °C [155]. The hydrogel, fabricated via external gelation with Ca^2+^, exhibited an orange-red to yellow color transition during storage. TVB-N levels in minced pork increased from 5.86 mg/100 g at day 0 to 15.3 mg/100 g at day 7, with the color change becoming visually apparent at day 6. A strong correlation was observed between the total color difference (ΔE) and TVB-N values (R = 0.848, R^2^ = 0.9816). However, the colorimetric response was influenced by relative humidity, with higher RH (98%) retarding the color change compared to lower RH (53%). Research has developed a multicolor plasmonic hydrogel capable of in situ formation of gold nanoparticles, serving as a full-history time-temperature indicator (FHTTI). Unlike conventional indicators, this two-dimensional FHTTI employs two mutually independent parameters—color and length—to separately indicate temperature and time, ensuring the accuracy and completeness of thermal-temporal information. The indicator successfully monitored time-temperature variations under complex variable-temperature conditions for over 30 days and is suitable for attachment to the packaging of temperature-sensitive products within supply chains to assess their efficacy [156]. Ding et al. prepared carboxymethyl chitosan (CMC) hydrogels via cross-linking with alginate derivatives (ADA). Leveraging the dynamic properties of Schiff base bonds and hydrogen bonds, these hydrogels exhibit self-healing capabilities. The incorporated anthocyanins undergo colorimetric changes upon exposure to acidic or alkaline gases, endowing the hydrogels with potential for application as smart indicators for detecting the freshness of chicken, pork, and fish in food packaging [157]. Tang et al. developed polyvinyl alcohol/sodium alginate (PVA/SA) hydrogels loaded with methyl red/bromothymol blue (MR/BB) as amine indicators for monitoring spoilage in beef, fish, chicken, and pork. The functional hydrogel was prepared through a combined freezing/thawing and calcium cross-linking process, optimizing sensitivity toward ammonia. Sigmoidal fitting results demonstrated that the RGB model integrated with a smartphone formed a strong correlation with total volatile basic nitrogen (TVB-N) values in meat products, enabling quantitative assessment of spoilage progression. This study validated the practical applicability of hydrogel-based colorimetric indicators for real-time food quality monitoring [158].

Additionally, a novel hydrogel humidity sensor has been developed using an acrylic acid/bagasse cellulose (AA/BC) porous hydrogel activated by cold plasma (CP). Both graphene oxide and citral were successfully loaded onto the AA/BC matrix, forming strong hydrogen bonds with the hydrogel. This system extended the shelf life of mangoes by approximately 8 days. By visually assessing the hydrogel sensor’s appearance and total color difference (TCD), its water absorption characteristics can be correlated with the citral release process, thereby enabling intelligent freshness preservation [159].

### 5.3. Food Quality Monitoring

Stimulus-responsive hydrogels, owing to their high water content, porous networks, and ease of functionalization, can respond swiftly to spoilage-related cues and convert these into intuitive, quantifiable output signals, serving as platforms for both food quality monitoring and intelligent controlled-release packaging [160,161]. In the context of food quality monitoring, hydrogel-based biosensors have been widely developed for the detection of various contaminants, including biotoxins, pesticide residues, antibiotic residues, pathogenic bacteria, heavy metal ions, and freshness indicators, leveraging their ability to immobilize biometric elements and facilitate signal transduction [160,161]. For intelligent controlled-release packaging, hydrogels enable the on-demand release of active substances such as antimicrobial agents in response to specific environmental triggers—including pH changes, temperature fluctuations, relative humidity, enzymes, or light exposure—thereby synchronizing preservation activity with food spoilage dynamics [160,161]. This approach optimizes antimicrobial efficacy and ensures food quality by releasing actives precisely when and where needed. Intelligent controlled-release antimicrobial packaging has been investigated for various food categories, including meat, dairy, and fresh produce, representing an innovative and challenging technology with significant potential for enhancing food safety and extending shelf life.

Colorimetric pH-sensitive hydrogel indicators integrated with smartphones have been developed for real-time visual monitoring of food spoilage. Tang et al. employed a polyvinyl alcohol/sodium alginate (PVA/SA) hydrogel as a carrier, loaded with the pH-sensitive mixed dye methyl red/bromothymol blue (MR/BB), which functions as an amine indicator. This hydrogel was successfully applied to monitor spoilage in beef, fish, chicken, and pork under different storage conditions. Sigmoidal fitting results demonstrated that the RGB model integrated with a smartphone formed a strong correlation with total volatile basic nitrogen (TVB-N) values in meat products, enabling quantitative assessment of spoilage progression [158].

Research has demonstrated the preparation of functional hydrogels by incorporating silver-doped Prussian blue nanoparticles (SPB) into agarose hydrogels. Trimethylamine (TMA) vapour permeates into the functional hydrogel matrix, triggering the decomposition of SPB nanoparticles. This process is accompanied by a colour change (from blue to colourless) and the disappearance of the photothermal effect. This approach successfully enables the assessment of shrimp/fish freshness [162].

A dual-functional hydrogel-polymersome composite (HPC) system has been developed by embedding dye-loaded polymersomes within a poly(N-isopropylacrylamide)-based hydrogel [163]. This platform enables temperature-triggered release of the encapsulated dye upon heating from 25 °C to 40 °C, while the released dye simultaneously serves as a pH-reporting molecule. The system achieved a detection limit of 1 mM methylamine for spoilage monitoring, demonstrating its potential as a multifunctional sensor for food packaging applications [163].

Real-time monitoring of volatile organic compounds (VOCs) released by perishable fruits is crucial for predicting their freshness. Kimia Esmaeili et al. designed a non-destructive, portable, hydrogel-based colorimetric freshness indicator. This colorimetric sensor array comprises a modified calcium alginate pad (CAP) coated with a mixed hydrogel of xanthan gum (XG) and rhamnan gum (TG), incorporating zinc oxide nanoparticles (ZnO-NPs) as a stabilizing filler. The array utilizes glycerol as a cross-linking agent and employs bromophenol purple (BCP), methyl orange (MO), BCP/thymol blue (ThB), and bromophenol green (BCG) as color-developing sensitive materials. It monitors acetone, acetaldehyde, and propionaldehyde markers in yellow fruits such as bananas, apples, and pears to assess fruit freshness [164].

### 5.4. Other

Stimulus-responsive hydrogels exhibit tunability and compatibility with extrusion-based printing techniques, whilst demonstrating structural and functional responses to external stimuli. Consequently, these hydrogels are applicable in 4D food printing [145].

Researchers have successfully developed a four-dimensional health food by combining an anthocyanin-potato starch (PS) gel with a lemon juice gel into a dual-component system. The 3D-printed anthocyanin-PS gel exhibits an enticing colour transformation over time (representing the fourth dimension). This colour transformation occurs not only when spraying the 3D product with solutions of varying pH levels, but also autonomously in response to pH stimuli originating from another component within the printed multi-material product [165].

To provide a more reliable curcumin stabilisation solution and efficient post-processing technique for food 4D printing systems, a study innovatively introduced nano-embedding technology and catalytic infrared drying (CID) methods to develop thermally responsive 4D-printed colour-changing materials. First, curcumin was encapsulated within whey protein isolate to form nanoparticles, which were subsequently incorporated into a potato starch-xanthan gum matrix to create 3D printing ink. Four-dimensional printed food products were prepared via catalytic infrared drying, microwave drying, and hot-air drying. A systematic investigation examined the effects of these drying processes on the physicochemical properties of the final products, including colour, texture, volume shrinkage rate, curcumin retention rate, and antioxidant capacity [166].

Stimulus-responsive hydrogels may also be employed for protein and lipid separation within the food industry. Whey protein constitutes a mixture of globular proteins, possessing high nutritional value and excellent functional properties. Ye et al. developed a dual-gated pH-responsive membrane with a heterogeneous structure for whey protein fractionation. By selectively modifying grafting sites, dimethylaminoethyl methacrylate (DMAEMA) and 4-vinylpyridine (4VP) were grafted onto the surface and within the pores of an ethylene-vinyl alcohol copolymer (EVAL) membrane, yielding the heterogeneous structure. The dual-gated structure of PDMAEMA and P4VP responds to pH variations, enabling multi-level regulation of membrane surface charge and sieving performance. Under a stepwise pH gradient from 3.0 to 8.0, a monolayer dual-gated pH-responsive membrane achieves primary fractionation of whey protein mixtures. This provides a sustainable solution for separating whey proteins from complex mixtures [167].

As reviewed by Tang et al., thermoresponsive poly(NIPAAm)-based hydrogels have been explored for protein separation applications, demonstrating size-selective transport behavior dependent on temperature-induced swelling transitions [168].

### 5.5. Performance Evaluation of Hydrogel Matrices

The selection of an optimal polymer matrix for fabricating stimuli-responsive hydrogels depends critically on the intended food application, desired response mechanism, mechanical requirements, and regulatory compatibility. Based on the literature reviewed in Section 4 and recent advances in the field, a comparative evaluation of the major biopolymer classes is presented below.

Polysaccharide-based hydrogels (e.g., chitosan, alginate, cellulose derivatives, pectin, κ-carrageenan) offer excellent biocompatibility, abundant functional groups for modification, and natural abundance. Chitosan exhibits intrinsic pH responsiveness due to its amino groups, making it suitable for targeted delivery in the gastrointestinal tract and for antimicrobial packaging [105,136]. Alginate enables rapid ionic gelation under mild conditions with divalent cations, facilitating encapsulation of probiotics and enzymes [112,150]. Cellulose derivatives (CMC, HPC, methylcellulose) provide tunable thermoresponsive behavior and mechanical strength, ideal for edible films and 3D printing inks [68,169]. A recent comprehensive review highlights that polysaccharide-based hydrogels can be classified into single (e.g., chitosan-based, alginate-based) and composite types (e.g., polysaccharide/polysaccharide, polysaccharide/protein, polysaccharide/nanomaterial), with composite hydrogels showing superior mechanical properties, stability, and controlled release capabilities compared to single-component systems [170]. Marine-derived polysaccharides (e.g., alginate, carrageenan, fucoidan) have gained particular attention for food applications due to their unique bioactivities including antioxidant, antibacterial, and immunomodulatory properties [171]. However, polysaccharide hydrogels often suffer from lower mechanical strength compared to synthetic polymers, which can be mitigated by blending or composite formation [68,84,170].

Protein-based hydrogels (whey protein, soy protein isolate, zein) combine nutritional value with stimuli-responsive behavior. They can form gels via heat, pH, or enzymatic crosslinking (e.g., transglutaminase) and are suitable for texture modification, bioactive delivery, and 3D food printing [115,119]. Whey protein-based microgels have been successfully used as fat replacers in low-fat cheese [116], while soy protein-polysaccharide composites exhibit excellent printability for dysphagia-friendly foods [172]. Recent research demonstrates that 3D printed emulsion gels stabilized by whey protein isolate combined with different polysaccharides (xanthan gum, guar gum, locust bean gum, gum arabic) can achieve β-carotene release rates exceeding 93% and bioaccessibility up to 33.95% during simulated digestion, with release mechanisms dominated by Fickian diffusion and framework erosion [119]. A novel zein-magnolol complex incorporated into carboxymethyl cellulose hydrogel films exhibited exceptional mechanical properties (elongation at break up to 206%, more than three times that of pure CMC films) and strong antioxidant activity (DPPH scavenging 42.41%, ABTS scavenging 97.39%), effectively extending the shelf life of fresh-cut jackfruit [173]. The main limitations of protein-based hydrogels include susceptibility to enzymatic degradation during storage and batch-to-batch variability, which require careful processing control [124].

Peptide-based hydrogels offer high programmability and biocompatibility, allowing precise tuning of gel properties through amino acid sequence design [125,126]. Self-assembling peptides derived from food proteins (e.g., soybean, whey) have shown promise for encapsulating bioactive compounds and for antimicrobial packaging. A recent breakthrough involves the development of a self-assembled antimicrobial peptide hydrogel from Pardaxin (a fish-derived peptide), which forms under mild conditions without chemical crosslinkers and exhibits excellent viscoelastic properties, shear-thinning behavior, and intrinsic antimicrobial activity against foodborne and drug-resistant pathogens through a ‘trap-and-kill’ mechanism [132]. This hydrogel demonstrates strong adhesion to meat surfaces, protection against Shewanella putrefaciens, UV barrier properties, and sustained release in liquid foods such as milk, with enzymatic digestibility ensuring food safety post-ingestion [132]. A comprehensive review emphasizes that peptide-based hydrogels possess adjustable properties including responsiveness to ionic strength, antimicrobial functionality for food preservation, and suitability for developing advanced food equipment such as 3D printers and encapsulation system [141]. However, their mechanical strength is often low, and large-scale production remains challenging, limiting current applications to niche areas [174].

Polyphenol-based hydrogels exploit the multifunctional properties of phenolic compounds—antioxidant, antimicrobial, and UV-blocking—making them attractive for active food packaging [138,141]. They can form dynamic networks through hydrogen bonding, hydrophobic interactions, and metal coordination, enabling self-healing and stimuli-responsive behavior [138]. Recent studies have demonstrated the development of active edible films by incorporating Opuntia cladode polyphenols into locust bean gum and mucilage matrices, achieving antioxidant activity up to 49.79% and effectively preserving apple slice freshness during 9 days of refrigerated storage by slowing ripening mechanisms and maintaining steady total soluble solids levels [175]. A polysaccharide-reinforced composite hydrogel encapsulating bioactive polyphenols from citrus peel waste achieved encapsulation efficiency of 85.74%, maintained >95% polyphenol retention for over 4 weeks at 35 °C, and released ~77% of encapsulated polyphenols after 9 h under simulated gastrointestinal conditions in a controlled, stimuli-responsive manner [176]. Yet, the stability of polyphenol-based hydrogels under varying humidity and temperature, as well as potential color changes, must be carefully managed for commercial use [137].

In practice, no single polymer is universally “best”; the optimal choice depends on the specific application requirements. For pH-triggered release in the gastrointestinal tract, chitosan and alginate are preferred [105,150]. For temperature-responsive food packaging, methylcellulose and gelatin are suitable due to their reversible gelation and GRAS status [75,169]. For 3D food printing, soy protein-polysaccharide composites and peptide-based hydrogels offer the necessary rheological properties and tunable functionality [119,120,130]. For antimicrobial packaging, peptide-based hydrogels (e.g., Pardaxin) and polyphenol-based hydrogels provide intrinsic bioactivity without requiring additional active agents [132,138]. Composite hydrogels combining two or more biopolymers often outperform single-component systems by synergistically enhancing mechanical strength, responsiveness, and functionality [84,96,170]. Therefore, a tailored design approach—matching the polymer matrix to the target stimulus and food matrix—is essential for successful application of stimuli-responsive hydrogels in the food sector.

## 6. Prospects and Challenges

Stimuli-responsive hydrogels offer transformative potential for food applications, yet their transition from laboratory prototypes to commercial products faces multifaceted hurdles. Below we critically examine these challenges across four interconnected domains and outline future directions.

### 6.1. Fundamental Material Limitations

Despite significant advances, the intrinsic properties of stimuli-responsive hydrogels impose fundamental constraints. Mechanical weakness remains pervasive—polysaccharide-based hydrogels typically exhibit compressive strengths below 100 kPa, whereas synthetic polymers exceed 1 MPa [81,82]. Composite strategies (e.g., incorporating cellulose nanocrystals or forming interpenetrating networks) can improve strength 3- to 5-fold [68,83], but often compromise responsiveness or increase complexity. Increasing crosslinking density enhances mechanical integrity but reduces swelling amplitude and slows response kinetics; for PNIPAM-based systems, raising crosslinker from 0.5 mol% to 2.0 mol% decreases equilibrium swelling by ≈40% [67].

Response specificity is another critical issue. Phenylboronic acid-based glucose sensors cross-react with fructose and galactose (binding constants 50–500 M^−1^ vs. 10–100 M^−1^ for glucose) [61], limiting their utility in sugar-rich matrices. pH-responsive hydrogels are suppressed by high ionic strength—swelling can be reduced by up to 60% in saline environments [22], making them unreliable in brined or fermented products. Multi-responsive systems introduce coupling effects that are difficult to predict; combined pH/temperature stimuli can alter the Flory–Huggins parameter by up to 20% [7], complicating rational design. These trade-offs between strength, responsiveness, and selectivity must be carefully balanced for each application [9,177].

### 6.2. Practical Application Barriers

Translating hydrogels from controlled laboratory conditions to real food environments reveals numerous practical limitations. Storage stability is a major concern—anthocyanin-based colorimetric indicators lose 35% of their response intensity after 30 days at 25 °C [155], while enzyme-responsive systems may undergo premature degradation due to residual proteases or humidity-induced hydrolysis during storage [51]. Humidity dependence further complicates deployment; color change in an alginate-methylcellulose indicator was delayed from 20 min at 53% RH to 24 h at 98% RH [153], illustrating the challenge of variable supply-chain conditions.

Light-responsive systems face penetration limits—attenuation coefficients for visible light in turbid foods exceed 10 cm^−1^, restricting effective activation to surface layers (<2 mm) [38]. Near-infrared light offers deeper penetration but requires photothermal agents that may migrate into food, raising safety concerns [44]. Moreover, photochromic molecules often fatigue, with >50% response reduction after 10 cycles [48]. For intelligent packaging, sensor responses must be validated across diverse products; a correlation established for pork spoilage (R^2^ = 0.98) [156] may not hold for fish or poultry, requiring recalibration for each application [11].

### 6.3. Regulatory and Safety Considerations

The regulatory landscape for stimuli-responsive hydrogels remains fragmented. Materials generally recognised as safe (GRAS) for conventional use (e.g., alginate, chitosan, gelatin) may face different standards when incorporated into active packaging where migration is intentional or unavoidable. The EU Framework Regulation (EC) No 1935/2004 requires that active materials not cause unacceptable changes in food composition, yet systematic studies on sensory impacts remain scarce [178].

Nanomaterial-containing hydrogels (e.g., silver-doped Prussian blue [160], graphene oxide [157], gold nanoparticles [154]) trigger complex questions about migration thresholds, chronic exposure, and environmental fate. Current frameworks were not designed for dynamic materials that change properties upon stimulation, creating regulatory uncertainty [178]. Enzyme-crosslinked systems require validation of residual enzyme activity; transglutaminase, widely used in processed meats, must be thermally inactivated to avoid post-processing modification of food proteins [93]. Each novel enzyme or genetically modified source demands case-by-case safety assessment, adding time and cost to approval [82].

### 6.4. Economic and Scalability Challenges

From an economic perspective, the commercial viability of stimuli-responsive hydrogels depends on balancing performance benefits against production costs. Recent techno-economic analyses of starch-based hydrogels estimate total capital investment of approximately 49.78 MMUSD with break-even prices of 4760.84 USD/t [179], while enzyme-crosslinked lignin hydrogels have a projected minimum selling price of 2141 USD/t [180]. Raw material costs dominate operating expenses, accounting for over 90% of total costs in polysaccharide systems. For food packaging applications, target unit costs of $0.03–0.06 per package have been identified for commercial viability [181]. Future cost reductions through process optimization and economies of scale will be essential for widespread adoption.

### 6.5. Future Directions

Addressing these challenges demands coordinated advances across materials science, process engineering, and regulatory science. Materials innovation should focus on simplifying multi-responsive systems—for instance, using single-polymer architectures with intrinsic multi-responsiveness rather than complex composites [13]. Process intensification (continuous manufacturing, enzyme immobilisation for catalyst reuse, integration with existing food processing lines) could reduce production costs by 30–50% while improving consistency [182].

Regulatory science must evolve to accommodate dynamic materials, developing standardised migration testing protocols that account for stimuli-triggered release. Collaborative initiatives between academia, industry, and agencies are needed to generate safety data without imposing prohibitive costs on innovators [178].

Ultimately, the successful translation of stimuli-responsive hydrogels from laboratory curiosities to commercial food technologies requires demonstrating clear value—whether through extended shelf life, reduced food waste, enhanced nutritional quality, or improved consumer safety—that justifies the additional complexity and cost. The field stands at an inflection point, where fundamental advances must now be coupled with rigorous systems-level thinking to realise their transformative potential [9,177].

## 7. Conclusions

Stimuli-responsive hydrogels represent a rapidly advancing frontier in food science, offering programmable materials that bridge dynamic food environments with precise functional delivery. This review has systematically analyzed the field across four interconnected dimensions: response mechanisms, fabrication strategies, matrix-specific design, and emerging applications. The behavior of these materials is rooted in well-established thermodynamic principles—Flory–Huggins theory, Donnan equilibrium, and free volume theory—which provide a unified framework linking molecular interactions to macroscopic swelling transitions and controlled release. Each response mechanism (pH, temperature, light, enzymes, glucose, multi-stimuli) offers distinct advantages and limitations for food applications. From a materials perspective, polysaccharides, proteins, peptides, and polyphenols each bring unique functional attributes—from the pH responsiveness of chitosan to the antioxidant activity of polyphenol-based networks. Composite systems synergistically combining these biopolymers often outperform single-component hydrogels, enabling tailored properties for specific food matrices. On the application front, significant progress has been made in targeted nutrient delivery, intelligent packaging, real-time quality monitoring, and 4D food printing. However, translation from laboratory prototypes to commercial products remains constrained by material limitations (mechanical weakness, response specificity), practical barriers (storage stability, humidity dependence), regulatory uncertainties, and economic challenges. Future success will require predictive materials design, process intensification, regulatory innovation, and systems-level integration with food supply chains. By coupling fundamental physicochemical understanding with practical engineering, stimuli-responsive hydrogels hold genuine promise to address pressing food safety, sustainability, and nutritional challenges.

## Figures and Tables

**Table 1:** Optimal formulation parameters for selected hydrogel systems.

Hydrogel System	Crosslinking Type	Key Parameters	Optimal Range	Performance Outcome	References
Poly (NIPAM)-based hydrogels	Free radical polymerization	NIPAM:comonomer ratio; crosslinker (MBA) concentration	Comonomer 0–20%; MBA 0.5–2.0 mol%	LCST tunable 25–40 °C; swelling ratio decreased by ~40%; elastic modulus increased from 10 to 45 kPa	[24,25,67]
SA-cl-PAA (poly(acrylic acid)-grafted sodium alginate)	Free radical polymerization	Crosslinker (MBA); initiator (KPS); monomer (AA)	MBA 1.5 wt%; KPS 0.8 wt%; AA 20 wt%	Maximum swelling 41.298%	[97]
PVME-based thermoresponsive hydrogels	Radiation crosslinking	Radiation dose	10–50 kGy	VPTT decreased by 3–5 °C; swelling ratio decreased by 25–35%	[72]
Self-healing hydrogels	Radiation crosslinking	Radiation dose	25 kGy	Healing efficiency up to 85%	[73]
Gelatin hydrogels	Physical (hydrogen bonding)	Polymer concentration	2–10% (*w*/*v*)	Melting emperature increased from 28 to 34 °C; elastic modulus enhanced ~10×	[75]
Methylcellulose hydrogels	Physical (hydrogen bonding)	Polymer conc.; salt addition	Tunable 40–60 °C (sol–gel)	Precise thermal gelation control	[9,98]
Hydrophobically associated polyacrylamide hydrogels	Physical (hydrophobic)	SDS:zwitterionic surfactant ratio	Optimized for ~690 kPa	Tensile stress ~690 kPa; self-healing	[81]
Alginate hydrogels	Physical (ionic)	Ca^2+^ concentration	50–75 mM	Optimal mechanical properties	[81,82]
Tyrosinase-crosslinked films (pea protein/chitosan/chlorogenic acid)	Enzymatic	PP:CS ratio; CA; tyrosinase	1:1; 20 mM CA; 18 U/mL Tyr	Tensile strength 130.78 MPa; DPPH 69.05%	[91]

## Data Availability

No new data were created or analyzed in this study.
